# Acceptability of a Culturally Tailored eHealth Video Intervention “Put Yourself First” to Promote Preexposure Prophylaxis Awareness and Knowledge Among Young Black Women: Mixed Methods Pilot Study

**DOI:** 10.2196/74132

**Published:** 2026-02-26

**Authors:** Keosha T Bond, Porche M Williams, Portia Thomas, David Babayev, Alana Gunn

**Affiliations:** 1 Department of Community Health and Social Medicine CUNY School of Medicine New York, NY United States; 2 Research Education Institute for Diverse Scholars Center for Interdisciplinary Research on AIDS Yale School of Public Health New Haven, CT United States; 3 School of Nursing Yale University Orange, CT United States; 4 Department of Criminology, Law and Justice University of Illinois Chicago Chicago, IL United States

**Keywords:** Black women, culturally tailored interventions, eHealth, health education, HIV prevention, PrEP, sex positive, stigma

## Abstract

**Background:**

Black women experience dual disparities with disproportionately high rates of HIV infection and low uptake of preexposure prophylaxis (PrEP). To address this issue, a culturally relevant eHealth video intervention was developed to increase awareness and uptake of PrEP.

**Objective:**

This study aims to evaluate the usability, acceptability, and impact of a culturally tailored eHealth video intervention (“Put Yourself First”) on PrEP-related knowledge and motivation among young Black women.

**Methods:**

This study used a sequential, mixed methods design. Thirteen young, primarily heterosexual, Black women, aged 18-25 years, were recruited from community-based organizations and social networks in New York City to participate in 2 evaluation focus groups. Participants completed pre- and postvideo assessments, followed by facilitated focus group discussions to elicit qualitative feedback on the video’s usability, acceptability, and impact on PrEP knowledge.

**Results:**

After watching the eHealth video, knowledge scores increased significantly postviewing (*P*=.04). Most participants indicated willingness to consider PrEP (10/13, 77%), and all reported intentions to recommend it to others (13/13, 100%). Overall, 92% (12/13) of the participants rated the video as very good or excellent. Participants found the video to be very interesting (11/13, 85%) and useful (12/13, 92%). The qualitative findings suggest that representation, authenticity, and the normalization of PrEP use emerged as critical drivers of acceptability. It also highlighted the video’s relatability, cultural relevance, and ability to dispel misconceptions, while also identifying areas for refinement for future studies.

**Conclusions:**

Findings support the acceptability and usability of culturally tailored sex-positive eHealth interventions for improving PrEP awareness and motivation among young Black women. Culturally resonant multimedia centering the voices of young Black women to affirming pleasure, agency, and addressing structural barriers to engagement offers a promising strategy for HIV prevention.

## Introduction

Despite Black women’s vulnerability to HIV transmission and the availability of preexposure prophylaxis (PrEP) in the United States, many Black women continue to lack essential information about its benefits and accessibility [1,2]. According to the Centers for Disease Control and Prevention (CDC), only a small percentage of eligible Black women were aware of PrEP, and even fewer were using it [3-6]. Cultural and structural factors, such as inadequate representation in PrEP advertising and provider discomfort discussing HIV prevention options with Black women, further hinder PrEP adoption [7]. Addressing these challenges requires culturally relevant messaging and targeted outreach to reduce multilevel barriers, such as limited functional knowledge of PrEP, low perceived susceptibility to HIV transmission, medical mistrust, stigma, and constrained health care access [5,7-12]. These efforts must also be aligned with empowering Black women to make informed decisions about their sexual health practices, including HIV prevention [13].

Research suggests that animated educational videos can be practical tools for improving health knowledge and health literacy across diverse populations [14-16]. These videos are generally well-received by participants and can raise awareness, facilitate engagement, and reduce anxiety [17-19]. Video-based interventions have been shown to increase not only knowledge but also comprehension, which reduces uncertainty in decision-making and improves patient outcomes compared to written material [19-21]. Multimedia formats, such as animation [21], and delivery channels, including mobile video messaging, have enhanced PrEP knowledge and increased comfort discussing PrEP with providers [22].

In the context of HIV prevention, video interventions have shown promise in increasing knowledge, awareness, and intentions to use PrEP among various populations, including Black women [23,24]. However, most video-based HIV prevention tools have been developed for men who have sex with men or broad, mixed-gender audiences. Black women’s prevention needs are shaped by gendered racism, relationship dynamics, and systemic health care inequities [4,25,26]; yet, few video-based HIV interventions have been explicitly designed for them related to PrEP [21,27]. Current eHealth interventions also tend to focus narrowly on nominal awareness, while neglecting relational and structural barriers, such as provider bias, partner mistrust, and burdensome medical monitoring that disproportionately shape Black women’s PrEP decisions [26,28]. A recent longitudinal study with young Black women also highlights that barriers to PrEP initiation change over time, emphasizing the need for ongoing support and integrated, culturally tailored PrEP education for women’s sexual health care [29]. Most HIV prevention messaging tends to focus on risk framing, while sex-positive narratives that affirm pleasure, intimacy, and autonomy are largely absent when directed toward women [30]. This common practice leaves a critical gap in approaches, perpetuates invisibility, and disengagement in HIV prevention efforts that could resonate more powerfully with the intersectional dynamics of Black women’s lived experiences [25].

The literature suggests that eHealth interventions, particularly video-based educational tools, can effectively increase awareness and knowledge about PrEP among Black women [21]. By addressing the current gaps in understanding and using engaging multimedia formats, there is potential to overcome barriers to accessing PrEP and ultimately improve health outcomes in this underserved population. The purpose of this study is 2-fold: (1) to evaluate the usability, acceptability, and impact of a culturally tailored eHealth video intervention (“Put Yourself First”) on PrEP-related knowledge and motivation among young Black women, and (2) to gather participant feedback to inform future refinements to its content and delivery. By directly addressing misconceptions, providing evidence-based information, and promoting open communication between providers and patients, this video aims to empower Black women to make informed decisions about their sexual health. Notably, the intervention uses culturally authentic narratives that are grounded in a sex-positive framework [31], positioning PrEP not only as an HIV prevention tool but also as a means of supporting autonomy, intimacy, and pleasure as integral aspects of women’s sexual well-being. This study was grounded in multiple theoretical frameworks that informed both the development and evaluation of the intervention. Specifically, intersectionality [6,32], Black Feminist Thought (BFT) [33], and the Theory of Triadic Influence (TTI) [34] helped identify multilevel barriers to PrEP uptake among young Black women, while the Information-Motivation-Behavioral Skills (IMB) model [35] guided mapping behavioral determinants of PrEP use. In addition, the education-entertainment (edutainment) framework [36,37] guided the translation of these theoretical insights into culturally tailored, sex-positive, and engaging video content. Collectively, these frameworks informed the design of a culturally tailored, sex-positive, action-oriented video intervention aimed at improving PrEP awareness and uptake.

## Methods

### Study Design Overview

**Figure 1:**
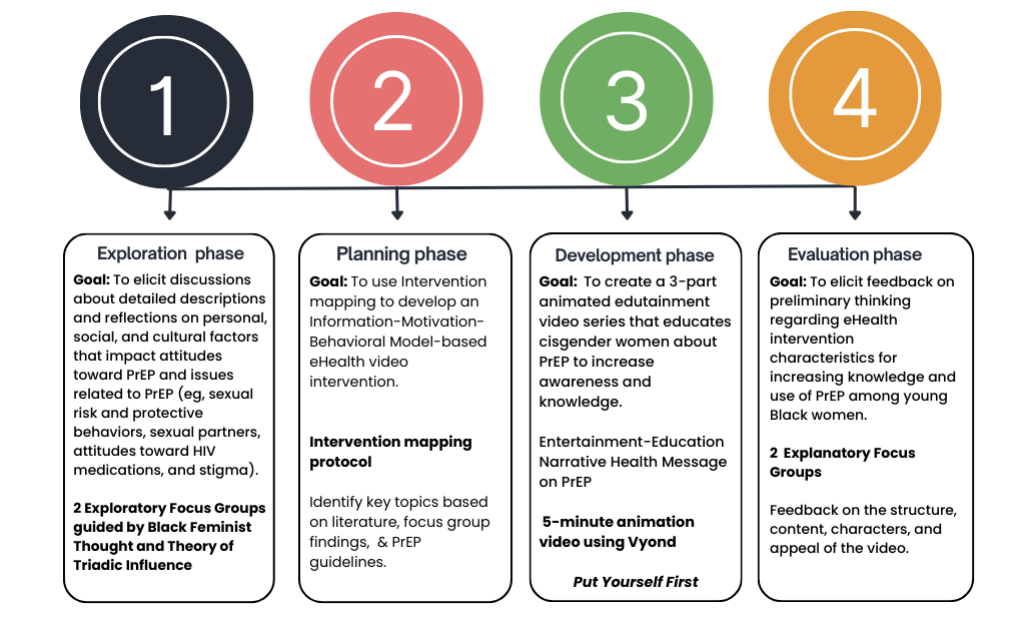
Project creation timeline for the “Put Yourself First” eHealth video intervention. PrEP: preexposure prophylaxis.

The LOVE (Learning Options through Video Education) study is a sequential, mixed methods pilot study designed to explore factors influencing Black women’s interest in oral PrEP and to assess the feasibility of a brief, culturally tailored eHealth intervention aimed at increasing PrEP awareness and motivation. Figure 1 depicts the study’s phased approach to developing and evaluating an innovative intervention. The four phases included (1) conducting exploratory focus groups to identify barriers and facilitators to PrEP uptake; (2) intervention mapping [38]; (3) development of the 5-minute, 3-part animated video, “Put Yourself First”; and (4) evaluation focus groups to assess the video’s usability, acceptability, and impact on PrEP knowledge. Detailed procedures for Phases 1, 2, and 3, including the literature review process and leadership of the intervention mapping activities, will be reported in a separate manuscript. This paper focuses exclusively on Phase 4, which evaluated the “Put Yourself First” video intervention.

### Study Sample

Women were recruited from community-based organizations and through social networking sites in New York City. The study recruited heterosexual Black women residing in New York City. The study prioritized this population due to the city’s disproportionately high HIV prevalence among Black women and the persistent gaps in PrEP awareness and uptake documented in prior research [39]. A member of the research team screened the women based on the following criteria: (1) aged 18 to 25 years; (2) self-identified as heterosexual; (3) self-identified as African American, Black, Caribbean Black, or multiethnic Black; (4) self-reported HIV-negative or with unknown HIV status; (5) reported oral, vaginal, or anal sex with a man in the past 12 months; (6) current or past problematic substance use; and (7) residency in New York City. We screened 78 women, of whom 37 (47%) were eligible and 26 (33%) consented to participate in the study. Reasons for nonparticipation after screening were not systematically collected. No nonparticipants (eg, observers or organizational staff) were present during the focus groups aside from the cofacilitators. Thirteen women from the final sample participated in the explanatory focus group discussions.

### Procedures

**Figure 2:**
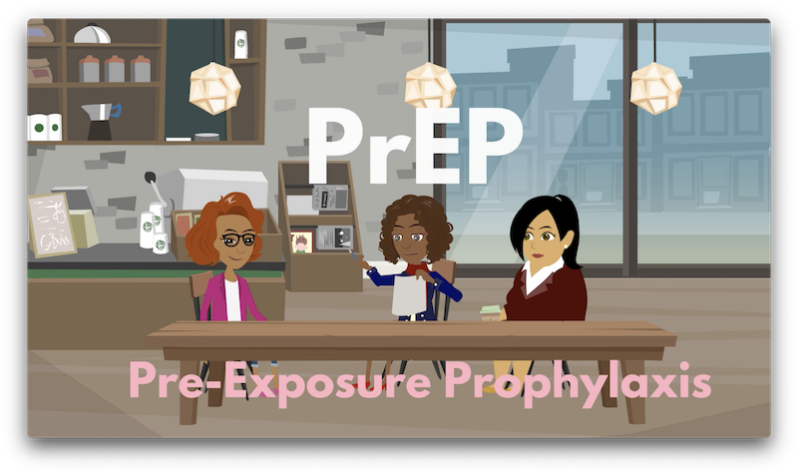
“Put Yourself First” video screenshot–animated scene introducing PrEP. PrEP: preexposure prophylaxis.

Study visits were conducted in English by trained research staff in a private space. The evaluation focus groups were co-led by the first author (a qualitative researcher with doctoral training in public health and extensive experience conducting focus groups with Black women) and a trained research assistant. Both facilitators were identified as Black women, which may have enhanced rapport and comfort among participants. Neither facilitator had prior personal relationships with participants. During recruitment and consent, participants were informed that the purpose of the groups was to gather honest feedback about the “Put Yourself First” video, and facilitators emphasized that there were no right or wrong answers. After rescreening for eligibility, participants underwent a detailed verbal informed consent process prior to data collection commencing. Eligible women were invited to participate in a Brief Assessment Battery (BAB) survey administered by tablet or paper and a focus group. The BAB validated measures to assess women’s initial knowledge of PrEP [40], HIV knowledge [41], sexual behaviors, relationship dynamics [42], self-efficacy [43], and HIV stigma [44]. During Phase 4, participants were recruited to participate in explanatory focus groups to evaluate the video’s effectiveness through 2 focus groups (Group 3: n=7; Group 4: n=6). The first part of the explanatory focus group examined the factors that impede and facilitate PrEP use [6]. Participants were shown the 5-minute educational video, “Put Yourself First” (Figure 2).

A still image from the eHealth intervention’s animated video shows 3 Black women characters engaging in a casual conversation at a café, introducing the topic of PrEP. The central character gestures while discussing PrEP, with the acronym prominently displayed in large white letters and the full term, Preexposure Prophylaxis, in pink text below. This visual design situates PrEP education within a relatable, everyday social setting, reflecting the intervention’s culturally tailored, peer-to-peer communication approach to normalize PrEP discussions among Black women.

After viewing the video, participants completed a postvideo survey to assess the acceptability, perceived impact, and effects on knowledge and intentions. The second part of the explanatory focus groups was conducted to elicit feedback on the video’s usability, structure, and appeal. The focus group guide included questions not only about the video’s content (accuracy, clarity of PrEP information, cultural resonance, and stigma reduction) but also about its format, style, and representation (eg, use of animation, relatability of characters, and overall usability and appeal). The guide ensured that participant feedback addressed both informational and stylistic components of the video. This article is centered on the section of the focus group discussion that addressed the video’s usability, structure, and appeal. The audio-recorded focus groups took approximately 2 hours to complete. Although field notes were not taken, audio recordings were transcribed verbatim to ensure accuracy. This paper was prepared in accordance with the COREQ (Consolidated Criteria for Reporting Qualitative Research) checklist to enhance transparency and rigor in reporting qualitative methods and findings [45].

### Postvideo Survey

The postvideo survey was administered immediately after participants watched the animated PrEP video (“Put Yourself First”). The postvideo survey comprises five parts: (1) PrEP knowledge (10 items, “True,” “False,” and “Don't know”) covering core facts such as HIV-negative eligibility, 3-month HIV testing while on PrEP, typical side effects, condom recommendations, distinction from postexposure prophylaxis, and hepatitis B virus considerations; (2) concerns about PrEP (13 items, “Yes,” “No,” and “Don’t know”) assessing safety (long-term and reproductive), side effects, resistance, perceived incomplete protection, daily pill burden, potential risk compensation (self and partners), stigma and privacy, comfort discussing sex with providers, and cost; (3) conditions for uptake (8 items, “Yes,” “No,” and “Don’t know”) eliciting enabling factors such as no-cost access, free HIV testing and monitoring, one-on-one or text-based counseling, group adherence support, and nonclinic access points; (4) acceptability, intentions, and global rating including willingness to recommend the video (Yes or No), a 1-6 overall quality rating, and intentions to consider or recommend PrEP; and (5) narrative engagement and perceived impact plus a brief global appraisal (5-point items) capturing story realism, message salience, character identification and liking, perceived social diffusion, and the likelihood of behaviors such as asking a provider about PrEP, requesting condom use, resisting unwanted sex, and obtaining HIV testing, alongside ratings of interest, usefulness, amount learned, comprehension, and liking.

### Data Analysis

Descriptive statistics from the surveys were calculated for the participants using IBM SPSS Statistics for Windows, version 29.0.2.0 [46], to summarize the data, including sociodemographic characteristics, sexual behavior, PrEP knowledge, and video acceptability. Pre- and posttest scores for PrEP knowledge were compared using paired-sample *t* tests for the full sample and within each group. Statistical significance was defined as *P*≤.05.

Audiotapes of the focus group discussions were transcribed. The transcripts were imported into Dedoose (SocioCultural Research Consultants, LLC) [47] software for coding and analysis. The research team analyzed the transcripts, but they were not returned to the participants for comment. Similarly, participants did not engage in formal member checking of findings, which we note as a methodological limitation. Two lead analysts and a research assistant, trained in qualitative methods, identified codes and used a multilayered strategy based on the topics addressed in the focus group guide. Analysis occurred in multiple stages, corresponding to the inductive approaches of open coding and axial coding used in grounded theory [48,49]. Codes were compared, refined, and reorganized through consensus discussions. To assess the breadth of issues raised, themes from the first focus group were compared to those from the second. While the small sample size precluded claims of definitive saturation, no substantially new themes emerged in the second group beyond those identified in the first. This consistency suggested adequate coverage of core issues for the scope of this exploratory evaluation [50,51]. Thus, we characterize our findings as reflecting preliminary thematic coverage rather than full saturation, consistent with grounded theory approaches in small-sample qualitative studies [52]*.* Here, we report evaluation findings from the 2 explanatory focus groups that assessed the eHealth video’s effectiveness and acceptability for increasing knowledge and addressing barriers to PrEP uptake among young Black women. All analysts worked independently to identify codes, ensuring that themes were consistently represented in the data.

### Ethical Considerations

This study was reviewed and approved by the New York University Institutional Review Board (approval number IRB-FY2017-408). All participants provided written informed consent prior to participation. Participants who completed the focus groups received US $45 in compensation and a 2-fare New York City Metrocard. To protect participant privacy and confidentiality, all data were deidentified, stored in a secure location, and securely transferred electronically to investigators involved in the analysis.

## Results

### Sample Description

Table 1 presents the sociodemographic characteristics of the participants in the explanatory focus group (N=13). Participants had a mean age of 22 (SD 2.4) years, and the majority were US-born (11/13, 85%). Over half (7/13, 54%) had completed high school or a General Education Diploma, while 38% (5/13) had some college or trade school education, and 8% 1/13) held a college degree; 38% (5/13) were current students. Most participants were unemployed (6/13, 46%) or worked part-time (5/39, 39%), with the majority (10/13, 77%) reporting an annual income of less than US $19,999. All participants had health insurance, and 62% (8/13) had a primary care provider.

**Table 1:** Description of LOVE (Learning Options through Video Education) study explanatory focus group participants (N=13).

Characteristics		Participants
Age (years), mean (SD)		22 (2.4)
**US-born, n (%)**		
	Yes	11 (85)
**Education, n (%)**		
	High school or GED^a^	7 (54)
	Some college or trade school	5 (38)
	College degree	1 (8)
**Current student, n (%)**		
	Yes	5 (38)
**Employment, n (%)**		
	Full-time	2 (15)
	Part-time	5 (39)
	Unemployed	6 (46)
**Annual income, n (%)**		
	Less than US $19,999	10 (77)
	More than US $40,000	3 (23)
**Health insurance, n (%)**		
	Yes	13 (100)
**Primary care provider, n (%)**		
	Yes	8 (62)
**Has children, n (%)**		
	Yes	3 (23)
**Relationship status, n (%)**		
	Committed relationship	4 (31)
	Dating	8 (62)
	Single	1 (8)
**Current HIV test (past year), n (%)**		
	Yes	9 (69)
**Positive STI^b^ results (lifetime), n (%)**		
	Yes	7 (54)
**Have main or steady partner, n (%)**		
	Yes	13 (100)
**Have sex with nonmain partners, n (%)**		
	Yes	5 (39)
**Main or steady partner has sex with other, n (%)**		
	Yes	5 (39)
**Did not use condom, 3 months, n (%)**		
	Main or steady partner	12 (92)
	Other partner (n=5)	3 (60)
**Knowledge of PrEP^c^, n (%)**		
	Yes	10 (77)
**Used PrEP, n (%)**		
	Yes	2 (15)
**Knowledge of PEP^d^, n (%)**		
	Yes	8 (62)
**Used PEP, n (%)**		
	No	13 (100)

^a^GED: General Education Diploma.

^b^STI: sexually transmitted infection.

^c^PrEP: preexposure prophylaxis.

^d^PEP: postexposure prophylaxis.

Regarding family and relationships, 23% of participants (3/13) had children, and most (8/13, 62%) reported being in a dating relationship, while 31% (4/13) were in a committed relationship. All participants reported having a primary or steady partner, and 39% (5/13) also engaged in sex with nonmain partners; notably, 39% (5/13) indicated their steady partner had other sexual partners. Condomless sex with steady partners was nearly universal (12/13, 92%) and occurred with 60% (3/5) of nonmain partners. Concerning HIV prevention, 69% (9/13) tested for HIV within the past year, and over half (7/13, 54%) reported having a sexually transmitted infection (STI) at some point in their life. While most participants knew about PrEP (10/13, 77%), only 2 (15%) had ever used it. Awareness of postexposure prophylaxis was lower (8/13, 62%), with no reported use.

### Pre- and Posttest of PrEP Knowledge

Table 2 presents the pre- and posttest knowledge scores for the participants by group and collectively. Across all participants (N=13), mean scores increased significantly from 4.6 (SD 2.9) at pretest to 6.5 (SD 1.8) at posttest, for a mean difference of –1.9 points (95% CI –3.7 to –0.14; t_12_=–2.3; *P*=.04). Group-level analyses indicated significant gains among Group 3 participants (n=7), whose mean scores improved from 4.2 (SD 2.9) to 6.3 (SD 2.1), a mean difference of –2.0 points (95% CI –3.3 to –0.69; *t*_6_= –3.7; *P*=.01). Group 4 (n=6) demonstrated improvement as well, with mean scores rising from 5.0 (SD 3.1) to 6.8 (SD 1.5), though the change was not statistically significant (mean difference –1.8; 95% CI –6.4 to 2.7; *t*_5_= –1.04; ns).

**Table 2:** Pre- and posttest PrEPa knowledge scores by group.

Group	N	Pretest, mean (SD)	Posttest, mean (SD)	Mean difference	95% CI	*t* test (*df*)	*P* value
Both	13	4.6 (2.9)	6.5 (1.8)	–1.9	(–3.7 to –0.14)	–2.3 (12)	.04
Group 3	7	4.2 (2.9)	6.3 (2.1)	–2.0	(–3.3 to –0.69)	–3.7 (6)	.01
Group 4	6	5.0 (3.1)	6.8 (1.5)	–1.8	(–6.4 to 2.7)	–1.04 (5)	ns^b^

^a^PrEP: preexposure prophylaxis.

^b^ns: not significant.

### Barriers and Facilitators to PrEP Uptake

Table 3 presents the key barriers and facilitators to PrEP uptake. Barriers to PrEP included concerns about side effects and stigma related to others noticing medication use, each reported by 62% (8/13) of participants, as well as concerns about reproductive health (7/13, 54%). Participants reported cost, daily pill burden, and partner expectations less frequently. Notably, no participants expressed discomfort about discussing sexual health with a provider. In contrast, strong facilitators were identified, particularly access to text-based support (12/13, 92%), counseling about sex and relationships (11/13, 85%), and free HIV testing, monitoring, and one-on-one counseling (10/13, 77%). More than half also reported that free medication (8/13, 62%) and group-based adherence support (7/13, 54%) would enable uptake.

**Table 3:** Barriers and facilitators to PrEPa uptake among young Black women postviewing (N=13).

Item		Participants
**Barriers, n (%)**		
	I am worried about the long-term effects of PrEP on my overall health.	6 (46)
	I am worried about the long-term effects of PrEP on my reproductive health.	7 (54)
	I am worried about the side effects.	8 (62)
	I am worried that if I do become HIV positive, certain medicines won’t work because I was taking PrEP.	2 (15)
	I am worried that PrEP does not provide complete protection against HIV.	5 (39)
	I am worried about having to take a pill every day.	2 (15)
	I am worried that taking PrEP might make me more likely to have sex without a condom.	4 (31)
	I am worried that having to take PrEP means I am putting myself at risk for HIV.	3 (23)
	I am worried that PrEP might make my partner(s) expect me to have sex without a condom.	1 (8)
	I am worried that people will see me taking medication and think I have HIV.	2 (15)
	I am worried that people will see me taking medication and will want to know why I am taking it.	8 (62)
	I am worried about having to talk to my doctor about my sex life.	0
	PrEP is too costly.	2 (15)
**Facilitators (I would take PrEP, if...), n (%)**		
	I did not have to pay for PrEP.	8 (62)
	I had access to free HIV testing.	10 (77)
	I had access to free sexual health and monitoring while on PrEP.	10 (77)
	I had one-on-one counseling and support around PrEP use.	10 (77)
	I had access to text-based support for PrEP use.	12 (92)
	I had access to support or counseling about my sex life.	11 (85)
	I did not have to go to my regular doctor to get PrEP.	4 (31)
	I had access to group-based adherence support for PrEP use.	7 (54)

^a^PrEP: preexposure prophylaxis.

### Video Acceptability and Perceived Impact

Table 4 presents findings on postviewing acceptability and intentions related to the PrEP video intervention. Most participants (10/13, 77%) reported they would consider using PrEP, and all (13/13, 100%) stated they would recommend it to others. The majority rated the video’s quality as very good (9/13, 69%) or excellent (3/13, 23%), and nearly all found it very or extremely interesting (11/13, 85%) and useful (12/13, 92%). Overall, 62% (8/13) reported learning a great deal of new information, while 92% (12/13) indicated they understood the content very well and liked the video very much.

**Table 4:** Postviewing acceptability and intentions of participants (N=13).

Item			Participants
**Intention to consider PrEP^a^, n (%)**			
	Yes	10 (77)	
Intention to recommend PrEP to others, n (%)			13 (100)
**Overall video quality rating, n (%)**			
	Very good	9 (69)	
	Excellent	3 (23)	
**How interesting was the video, n (%)**			
	Very interesting	5 (39)	
	Extremely interesting	6 (46)	
**How useful was the video, n (%)**			
	Very useful	6 (46)	
	Extremely useful	6 (46)	
**Amount of new information learned from video, n (%)**			
	None	2 (15)	
	Little	1 (8)	
	Some	2 (15)	
	A great deal	8 (62)	
**How much did you understand in this video, n (%)**			
	Very much	12 (92)	
**How much did you like the video, n (%)**			
	Very much	12 (92)	

^a^PrEP: preexposure prophylaxis.

Table 5 presents data on narrative engagement and the perceived impact of the PrEP video. Most participants found the storyline credible (9/13, 69%) and agreed that the message raised awareness (10/10, 77%) and supported action (8/13, 62%). Overall, 62% (8/13) reported relating to some characters and 31% (4/13) to most or all of them. The main character was universally well-received, with all participants describing her as likable, relatable, and compelling at emphasizing key messages. In terms of behavioral intentions, participants expressed a high likelihood of obtaining HIV testing (11/13, 85%) and asking a provider about PrEP (10/13, 77%), with a moderate likelihood of recommending PrEP (8/13, 62%), requesting condom use (7/13, 54%), and resisting condomless or unwanted sex (6/13, 46%).

**Table 5:** Narrative engagement and perceived impact of the video (N=13).

Item		Participants
**Story realism or credibility, n (%)**		
	Definitely Yes	9 (69)
**Message raises awareness, n (%)**		
	Definitely Yes	10 (77)
**Message supports action, n (%)**		
	Definitely Yes	8 (62)
**Identification with characters, n (%)**		
	I could relate to some of the characters.	8 (62)
	I could relate to many of the characters.	1 (8)
	I definitely related to most or all the characters.	4 (31)
**Main character, n (%)**		
	Likeability	13 (100)
	Type of friend you like to talk to	13 (100)
	Emphasize important messages	13 (100)
	Add to experience of learning about PrEP^a^	11 (85)
**Likelihood, n (%)**		
	Ask a provider about PrEP	10 (77)
	Recommend PrEP to someone	8 (62)
	Request condom use	7 (54)
	Change attitude about having unwanted sex	6 (46)
	Resist condomless sex	6 (46)
	Obtain HIV testing	11(85)

^a^PrEP: preexposure prophylaxis.

### Feedback From Explanatory Focus Groups

The qualitative findings identified 6 areas of focus: viewers’ engagement and perceived impact, relatability through authentic representation, navigating relationships while prioritizing personal health, structural and practical barriers to PrEP adherence, empowerment through health autonomy, and domains requiring further development.

#### Viewers’ Engagement and Perceived Impact

Participants found the video engaging and informative, describing it as “cute” and compelling in its provision of critical information about PrEP. The participant said, “It’s a nice way to get information out.” For some participants, the video served as a valuable educational tool by introducing new information about PrEP’s availability for women. As 1 participant noted, “It gives you more of that knowledge and info, so you understand it a little bit more.” The video successfully addressed the misconception that PrEP is only for gay men, reinforcing its relevance for women. They appreciated how it addressed and clarified misconceptions, such as the belief that PrEP is only for gay men. As 1 participant shared, “It helps with the message because the main person that was talking was a Black lady and she was just basically letting them know it's not only for gay people and you know, whatever the case may be. And it's good for women to take it because it helps as an addition to the condoms, you know.” Many participants described the video’s message as powerful and informative, providing a better understanding of PrEP’s purpose and its role in preventing HIV. Participants valued the emphasis on PrEP being a personal choice, with one saying, “powerful message,” and another adding, “It gives you more of that knowledge and info so you understand it a little bit more.”

#### Relatability Through Authenticity

Participants found several scenarios in the video particularly relatable to their own experiences. Many appreciated the conversational style, noting the realistic portrayal of 3 friends casually discussing PrEP over coffee, mirroring how “home girls” would talk among themselves, which added credibility to the message. One participant stated, “It was just a conversation between 3 home girls. I say home girls having coffee or whatever. You know it wasn't. Well, of course, it's scripted, but it didn't feel scripted. It felt like you were just watching like three characters having a normal conversation.” Another participant noted that the friends’ reactions to PrEP in the video reflected how she expected her peers to respond, with some being receptive and others skeptical. “I feel like that's how my friends would react, the way her friends were reacting. You know you're trying to tell people things to help them, especially your friends, but everybody has their own opinion and everybody [does] things on their own time.” Participants emphasize how the characters engaged in a mutual exchange of information about PrEP, making the learning process feel organic and authentic. As 1 participant highlighted the collaborative and peer-driven learning aspect of the video, “The good thing was that all three of them [were] sharing information about PrEP to each other. They were actually learning from each other.”

#### Navigating Relationships While Prioritizing Personal Health

Many participants resonated with the challenge of balancing personal health decisions with concerns about how a partner might perceive PrEP use, including fears of mistrust or judgment. A discussion centered around how introducing PrEP might raise questions in a relationship: “It probably would raise questions, but at the same time, then it's like, you know, why are you taking this all of a sudden?” A recurring theme in the discussion was relationship dynamics and the tendency for Black women to put others’ needs before their own, particularly in relationships, making it difficult to prioritize personal health. The scenario where a character contemplates her partner’s response to her using PrEP resonated deeply, reflecting common experiences among participants. One participant highlighted this dynamic, stating, “One of the characters was like, 'What about what he thinks,' and she was like, 'That's the problem with Black women. They always put people first before themselves.' I closely identify with that, but I never really thought about it. I think that's one of our biggest traits.” Another participant echoed this sentiment, emphasizing the tendency to prioritize a partner’s feelings over personal health: “She's not thinking about herself first. She's thinking about what he's going to think.” Concerns about a partner’s perception of PrEP use also emerged, as one participant explained, “I would say letting her partner know. I mean, like, to be honest, for some women, they would feel some type of way because you're taking something to prevent them, so probably the man that she's with is going to feel like she's doing something.”

#### Structural and Practical Barriers to PrEP Adherence

Participants found the video’s depiction of a character’s reluctance to adhere to the medical visits required for PrEP highly relatable and honest. Many agreed that frequent doctor visits pose a structural barrier to adherence, as women often juggle multiple responsibilities, making it difficult to schedule regular health care appointments. To improve accessibility, some participants suggested including alternative options, such as home testing, in the messaging. One participant emphasized this point, stating, “The biggest issue is the inconvenience of having to go to the doctor. A home test would make a big difference. Along with your prescription, you get a home test.” The video also ignited a discussion on the varied perspectives surrounding adherence and health care engagement. While some participants expressed a strong sense of personal responsibility in maintaining their health, others highlighted the challenges of staying consistent with medical visits. One participant explained, “I say it could, but it depends on who you are. Some people like to keep up with themselves. Like me, I'm that type of person. Like, I don't want anything to happen to me. I'm overprotective. I try to protect myself from a lot of stuff, you know. You only get one life. So, some people are different.”** **However, others acknowledged that even if someone is committed to taking PrEP, maintaining regular medical visits can be difficult. Another participant noted, “I think some people may just take the medication but just not go to the doctor, as they’re supposed to. It’s not to say it's going to stop them from taking the medicine, but they just might not go to the doctor every three months like they're supposed to.” This suggests that the video could further emphasize solutions for making healthcare more accessible and convenient.

Additionally, the conversation explored the practicability of taking PrEP alongside birth control, a common concern among participants. Some felt confident in their ability to manage both medications, with one stating, “Fine, because if you can remember to take your birth control pill every day, you're going to remember to take the PrEP too.”** **However, others pointed out the challenges of maintaining a daily medication routine. One participant shared, “I always miss my birth control pills because I have alarms. Sometimes I'm like, 'Oh my god. It's nine o’clock,' and I miss my pill. I'm not really good with pills, to be honest. I'm not really good with it, but I try my best or I'll ask somebody else to remind me. But if it’s important like that, I feel like it should be a priority.”** **This suggests that the video could acknowledge real-life struggles with adherence and offer practical strategies for staying on track. Beyond forgetfulness, concerns about misuse also emerged, with some participants expressing worry that individuals might treat PrEP similarly to birth control, believing they could compensate for missed doses or use it as a justification for engaging in riskier behaviors.

One participant explained, “I kind of just look at this stuff like, just like the birth control pill, women who use the birth control pill will use the PrEP. But I feel like people would be less responsible as far as, like, with the birth control pill. 'Oh, I just missed a day. I can just double up this day,' or 'I could have unprotected sex, but I know if I just take the pill...' or 'If I take the PrEP, if I double up this day, I can have unprotected sex this day.' People will abuse it like they abuse everything else.”

This concern suggests that the video could benefit from reinforcing clear messaging about proper PrEP adherence and dispelling misconceptions about its use.

#### Empowerment Through Health Autonomy

The video’s message about taking PrEP as an act of self-care resonated strongly, reinforcing the importance of prioritizing one’s health over others’ opinions. The main character in “Put Yourself First” emphasized taking PrEP for oneself rather than for others. Participants recognized the significance of making their own health decisions without feeling pressured to seek validation or approval from others, especially their partners. One participant said, “I just don't feel like he needs to know that. It's going to lead to another conversation. I don't think he needs to know everything. They don't need to know every detail because they don't tell us every detail.” This quote underscores the belief that women should have autonomy over their health choices and should not feel pressured to disclose every aspect of their health care decisions. Another participant reinforced this perspective, asserting, “You don't have to tell your partner about it,” highlighting that PrEP use is a personal choice and women should feel empowered to take control of their sexual health on their own terms. Affirming this theme of self-determination, another participant stated, “You're doing it for yourself at the end of the day,” further emphasizing the message that prioritizing one's health is an act of self-care and autonomy. Overall, these scenarios and messages were praised for authentically representing the realities and concerns of young Black women considering PrEP. Participants particularly valued the framing of PrEP as an act of self-care and empowerment, resonating with sex-positive narratives that affirm women’s right to prioritize both protection and pleasure in their intimate lives.

#### Domains Requiring Further Development

Table 6 outlines the domains identified by the participants that require further development to enhance the educational video about PrEP for future research. While participants found the video informative, they identified areas for enhancement to increase its relatability and impact. Participants suggested making future videos more relatable by incorporating real-life characters and scenarios to enhance impact and engagement. Participants suggested incorporating more storylines that reflect women’s daily lives and strategies for addressing the challenges and barriers to PrEP use, as well as adding depth to character interactions to make the video more engaging. They recommended replacing animations with real women to create a stronger emotional connection and enhance trustworthiness. While participants appreciated the inclusion of details about PrEP, they expressed concerns that highlighting side effects, such as nausea, could discourage viewers. They suggested presenting sufficient information to educate without overly emphasizing negative aspects, ensuring the benefits of PrEP remain central. Overall, the recommendations aim to enhance the video’s realism, solution-oriented approach, and impact by equipping the target audience with the skills necessary to address challenges related to accessing and maintaining PrEP adherence.

**Table 6:** Suggestions for improvement from explanatory focus groups (N=13).

Area of focus	Critical feedback	Suggestions for improvement	Illustrative quotes
Scenario	Realism and relatability: While the video was informative, but it lacked the realism needed to resonate with the target audience fully.	More realistic storylines: Participants suggested adding a storyline that more authentically reflects women's daily lives and challenges to make the video feel more genuine and engaging. Adding more depth to the character dynamics and their interactions could make the video feel even more realistic.	“Have people bring up past experiences. Have people have testimonies and that will get to people as people know that people have been through stuff and it's not just them and they could get through it or they can take this and be better or protect themselves better.”
Characters	Character relatability: While the characters conveyed the message well, some participants felt the group’s dynamic was a bit unusual.	Using real characters: Participants wanted to see real women (humans instead of animation) discussing PrEP^a^, as they felt this would make the message more trustworthy and more straightforward to connect with.	“I think maybe a...With actual, real characters. Maybe like a little scenario. You see how they were there but something a little more realistic. Like a little story line. That would reach more people and grasp their attention more, but this was nice. It was informative but it was a little silly. I think if you use a real scenario with real people, it might reach a greater population.”
Content	Side effects mentioned: Some participants felt that discussing potential side effects, like nausea, could be off-putting or scare potential users.	Balancing details: Participants wanted enough information to be well-informed but felt that too many details, especially about side effects, might discourage viewers from considering PrEP.	“If you go into any more details, you're gonna start scaring people.” “It was enough because then you talked about having to have kidneys checked and stuff like that. When you start talking about people got to get their kidneys checked, they are going to question,” what is this going to give to me?” Because your kidneys are very important.”

^a^PrEP: preexposure prophylaxis.

## Discussion

### Principal Findings

This study evaluated “Put Yourself First,” a culturally tailored, sex-positive, animated eHealth video intervention designed to increase PrEP awareness among young Black women. Participants found the video to be engaging, relatable, and informative, reporting increased knowledge of PrEP, a heightened willingness to consider it, and strong intentions to recommend it to their peers. Participants’ feedback suggested the importance of representation, authenticity, and the normalization of PrEP use for women, indicating the need for culturally resonant multimedia to enhance HIV prevention messaging. However, they also identified several aspects that could be refined to enhance its impact and resonance with the target audience.

Focus group findings demonstrate that the video was both engaging and educational, with participants praising its clarity, appeal, and ability to dispel misconceptions, such as the notion that PrEP is exclusively for gay men, while emphasizing its relevance and safety for women. These results align with prior research highlighting the importance of culturally tailored and gender-specific messaging in increasing PrEP awareness among women [8,53,54]. Importantly, these findings extend earlier exploratory studies [21] by demonstrating that an intervention explicitly grounded in sex-positive and empowerment frameworks can enhance knowledge while also resonating emotionally with the target audience. Previous research has shown that interventions can be enhanced by improving communication about sexual health within a young Black woman’s social network, particularly with emotionally supportive friends and family [55]. The conversational format among friends was viewed as authentic and relatable because it included a peer-to-peer exchange that mirrored participants’ real-life discussions. In edutainment, content is presented in ways that feel authentic and familiar, such as the script of the video, which conveys information on PrEP through a conversation among friends [37]. This educational method allowed the participants to see themselves or people like them. When people see peers (or peer characters) discussing, modeling, or demonstrating healthy behaviors, it helps participants believe that the behavior is possible, socially acceptable, and relevant [37]. The peer-to-peer exchange also models collectivism among Black women and communities [54].

At the same time, women identified relational challenges, particularly the tension between prioritizing their own health and concerns about partner perceptions, which reflects broader gendered and racialized dynamics that influence sexual health decision-making among Black women [6,55-57]. This reflects intersectional dynamics that Black women experience [6,55], where their overlapping identities, gendered expectations, and experience of racialized stigma impact structural inequities and constrain women’s HIV prevention options. Finally, the video’s framing of PrEP as an act of self-care and empowerment resonated strongly, affirming women’s right to make autonomous decisions about their sexual health. This finding contributes to the literature on sex-positive and empowerment-based approaches to HIV prevention, emphasizing the importance of interventions that affirm pleasure, center health autonomy and agency rather than relying solely on risk framing [58,59]. Together, these insights suggest that narrative-based, culturally resonant, and empowerment-framed interventions may be particularly effective for increasing PrEP engagement among Black women.

Although the video has notable strengths, participants noted several challenges that could limit its acceptability. While the video was informative, some participants found the use of animation somewhat limiting in fostering deep emotional connection and trust. They suggested incorporating real-life testimonials from women who use PrEP, live-action scenarios, or documentary-style storytelling on topics such as conversations with partners, health care visits, or navigating daily life while on PrEP, to enhance authenticity and relatability in PrEP-focused edutainment content [60]. There was a strong desire to see more authentic scenarios depicting real challenges women face when discussing PrEP with partners and health care providers, such as fear of judgment, accusations of infidelity, or adverse partner reactions. This aligns with prior studies highlighting the central role of interpersonal stigma and relationship dynamics in shaping women’s PrEP decision-making [6]. While women view PrEP as a tool for maintaining health and relationships, women’s romantic feelings and expectations can affect their risk perceptions and interest in using PrEP [61]. Imbalanced relationship dynamics, including power struggles, infidelity concerns, and trust issues, significantly influenced women’s PrEP decisions [62]. In addition, framing PrEP within a sex-positive, empowering context that aligns with women’s reproductive values may increase uptake [31,63]. These strategies not only enhance authenticity but also align with principles of edutainment, where culturally resonant narratives and modeled behaviors transform health information into engaging, meaningful content that promotes learning and supports behavior change.

Along with increasing awareness, participants emphasized the importance of directly tackling the institutional constraints that influence women’s PrEP decisions. They described persistent challenges, such as PrEP stigma, frequent medical visits, medical mistrust, and challenges with daily pill adherence, which often parallel their experiences with birth control [64-66]. Although the video demonstrated PrEP’s effectiveness, it did not fully address structural concerns, such as navigating the health care system, financial accessibility, or alternative care models, including pharmacy-based PrEP access or telehealth services [67-69]. Taken together, these findings emphasize the importance of advancing practice innovations that reduce both structural and interpersonal barriers through accessible care models, stigma-free environments, and culturally responsive provider communication. To enhance the impact of future interventions, messaging should include practical strategies to overcome these barriers, such as highlighting mail-order PrEP services, home HIV or STI testing kits, and financial assistance programs [68]. At the same time, future research is needed to evaluate multilevel strategies that address these barriers and generate evidence on how best to sustain equitable PrEP uptake among Black women. Such efforts will be crucial to ensuring that PrEP implementation not only increases awareness but also translates into meaningful improvements in HIV prevention outcomes [69]. Finally, these findings suggest the importance of expanding insurance coverage, integrating PrEP into reproductive and primary care services, and funding community-based initiatives that directly address the structural inequities limiting Black women’s access to HIV prevention [70].

In addition to addressing these structural barriers, it is crucial to consider how the “Put Yourself First” video can be disseminated most effectively. The findings emphasize the importance of designing HIV prevention interventions that not only raise awareness but also facilitate direct connections to services. Future dissemination strategies should leverage trusted community-based organizations, college health centers, sexual and reproductive health clinics, and digital platforms (both social networks and institutional services) to maximize reach and engagement among young Black women [9,10,13]. To ensure that the awareness generated by the video translates into tangible access to PrEP-related services and strengthens its real-world impact, direct referral pathways could be embedded within these dissemination channels, such as integrating links to PrEP locators, telehealth services, or peer navigation programs [54,67-69]. By pairing education with actionable referral tools, the intervention can more directly support women in moving from interest to uptake. These strategies can help bridge the persistent gap between PrEP awareness and actual uptake, ensuring that dissemination efforts are linked to concrete service access and continuity of care [26,28].

### Limitations

Several limitations warrant consideration. The findings are based on a small sample of young Black women from a single urban location, which limits their generalizability. Future work should extend recruitment to multiple geographic regions, including the South and rural communities where HIV disparities are most pronounced. Black women’s experiences are diverse and shaped by intersecting factors such as age, sexuality, and socioeconomic status. Future studies should purposively sample across these intersecting identities to capture heterogeneity within the population. While knowledge gains and acceptability outcomes were encouraging, the findings should be interpreted with caution, as they were not uniformly significant across all groups (eg, Group 4). Participants continued to express concerns about side effects, daily adherence, and structural barriers, such as frequent medical visits and medical mistrust. The evaluation was limited to immediate outcomes and did not assess downstream behaviors such as PrEP initiation or adherence. Participants also explicitly called for greater realism through live-action or testimonial-based storytelling and requested deeper engagement with structural barriers such as stigma, provider bias, and medical mistrust. These mixed findings temper the conclusions and highlight the need for future studies to address both behavioral and structural outcomes in addition to knowledge and acceptability. Longitudinal and controlled studies are needed to evaluate whether such interventions increase actual PrEP uptake and adherence. By incorporating these refinements, future studies can better bridge the gap between pilot findings and real-world application, ensuring that dissemination strategies not only raise awareness but also facilitate access and sustained use of PrEP.

Although no new themes emerged after the second focus group, the small qualitative sample limits the claim of saturation. Focus group methodologists typically recommend 3-5 groups per relatively homogeneous population to ensure adequate coverage of perspectives, suggesting that saturation should be monitored across, rather than within, groups [71,72]. While Guest and colleagues [73] demonstrated that most themes emerge within 6-12 participants, a benchmark often used to justify 2-3 focus groups for achieving saturation. This analysis of the explanatory focus groups aligns with this range, as it is a relatively homogeneous sample and the study consisted of targeted research questions. Additionally, focus group settings may have introduced social desirability bias, potentially leading participants to overemphasize favorable responses about the video. This study did not include specific methodological steps recommended by COREQ. For example, reasons for nonparticipation were not collected, field notes were not taken during focus groups, and transcripts were not returned to participants for validation. While focus groups were co-led by trained facilitators who shared social identities with participants, reflexivity was addressed through consensus-based coding discussions rather than participant checking of themes. These omissions may have limited opportunities to triangulate interpretations or further ensure the credibility of findings. Despite these limitations, the study provides critical insights into the acceptability and impact of a culturally tailored, sex-positive eHealth intervention video, offering a valuable foundation for refining and scaling PrEP promotion strategies for young Black women. This study reinforces existing research that demonstrates the effectiveness of culturally relevant, video-based educational interventions in improving engagement and increasing knowledge of PrEP among Black women [21,27,74].

### Conclusion

This study highlights the critical need for culturally tailored interventions to enhance PrEP awareness and uptake among young Black women. This research emphasizes the importance of using sex-positive, engaging, and culturally affirming sexuality educational tools to address the ongoing barriers of stigma, misinformation, and health care inequities. By embedding PrEP within sex-positive and empowerment frameworks, this study contributes novel evidence that HIV prevention can be framed not only as risk reduction but also as a pathway to autonomy, intimacy, and pleasure. The findings show that eHealth interventions, especially edutainment animated videos, can effectively raise awareness, dispel misconceptions, and encourage informed decision-making regarding PrEP. These findings will guide future iterations, examining the long-term effects of eHealth video interventions on PrEP uptake and adherence and ensuring that dissemination strategies extend beyond awareness to support real-world access and sustained PrEP use. Ultimately, this study emphasizes the transformative potential of culturally competent and innovative health communication strategies in tackling disparities and empowering Black women to take control of their sexual health. Centering women’s voices, addressing structural barriers, and affirming pleasure and agency must remain central as PrEP interventions continue to evolve.

## Abbreviations

BAB: Brief Assessment Battery
BFT: Black Feminist Thought
CDC: Centers for Disease Control and Prevention
COREQ: Consolidated Criteria for Reporting Qualitative Research
IMB: Information-Motivation-Behavioral Skills
LOVE: Learning Options through Video Education
PrEP: preexposure prophylaxis
STI: sexually transmitted infection
TTI: Theory of Triadic Influence
